# The paradox of sleep quality and training load in deaf handball players? An investigation of weekly changes and relationships

**DOI:** 10.1186/s13102-025-01443-5

**Published:** 2026-01-02

**Authors:** Mustafa Furkan Ocak, Zeki Akyildiz

**Affiliations:** 1 Sports Science Faculty, Training and Movement Science Department, Gazi University, Ankara, Türkiye; 2 Sports Science Faculty, Department of Coaching Education, Afyon Kocatepe University, Afyonkarahisar, Türkiye

**Keywords:** Training load, Sleep quality, Handball, Monitoring

## Abstract

**Purpose:**

This study examines (i) the relationships between training load and sleep quality parameters of deaf handball players and (ii) their weekly differences. Athletes went through four different camp periods in 45-day camp periods.

**Methods:**

Sleep quality and training load data were obtained from athletes in four separate camp periods, each lasting 10–12 days. Spearman correlation analysis was performed to determine the relationship between Training Load and Sleep Quality. Kruskal–Wallis test was used to determine the differences in sleep quality and training load parameters between blocks. Dunn’s post hoc analysis was performed to determine which block caused the differences.

**Results:**

As a result of the analyses, there are statistically significant differences between both training load and sleep quality between weeks and between weeks in terms of sleep falling asleep and sleep quality [How long did it take to fall asleep (minutes) (*p* = 0.001: η²p: 0.057)]. As a result of Spearman correlation analysis, no statistically significant relationship was detected between training load and sleep quality parameters. No statistically significant relationship was found between the sleep quality of handball players and their training load values. There are statistically significant differences in sleep quality and training load values between weeks.

**Conclusions:**

The analysis reveals statistically significant differences in training load and sleep quality between weeks, as well as between the times of falling asleep and sleep quality within weeks.

## Background

The concept of general adaptation syndrome is considered in training for athletes to achieve optimal performance [1]. The general adaptation syndrome aims to ensure progressive development in athletes by creating stress in sufficient doses during training [1]. To manage the process by determining the appropriate doses in training, it is necessary to assess the stresses on the organism [2–4]. For this reason, the dose-response system is considered in the training management [5, 6]. The stresses caused by training on the organisms of athletes are called doses [7]. There are responses to the stress on the organism through training [3, 6, 8, 9]. In recent years, the responses obtained from doses are commonly expressed with the term “training load” [3, 6, 10–12]. At this point, various methods have been developed by sports scientists and coaches to record the responses [3, 10]. Rating perceived exertion (RPE) is one of the most common methods of tracking training load. It is a method that does not require equipment or cost; only subjective questions are asked of the athletes [13–16]. After the training, the total load of the training is determined by multiplying the value obtained from the RPE by the duration of the training [14].

Training load detection optimizes sporting performance, but is insufficient [3, 10]. Several factors are available, including various recovery strategies after training, nutritional supplements, and the amount of sleep [17]. Among these multiple factors, sleep duration and sleep quality have been discussed in various studies over the past few years [18–24]. Sleep quality is essential for athletes to recover adequately [18, 19]. Sleep status before and after activities affects sports performance [18–21]. The duration and quality of sleep play critical roles in optimal performance [19, 24]. Sleep quality is of great importance not only for sports performance, but also for health [23, 24]. Studies have shown that a decrease in sleep duration and quality is associated with an increase in athletes’ injury rates [19].

Furthermore, research on the effects of training load on sleep quality and duration has been conducted in various populations [18–24]. In another study on training load and sleep quality, it was observed that as the training load varied from week to week, changes in sleep quality occurred at the same rate as the training load [20–22]. Several studies have examined training load, recovery, and sleep quality [19, 23, 24]. These studies are essential for sports scientists and coaches to organize their athletes according to scientific results. However, to the authors’ knowledge, no previous research has examined the relationship between sleep quality and training, as well as the weekly differences in sleep quality among deaf handball players. Therefore, this study aims to explore the (i) relationships and (ii) weekly differences in the training load and sleep quality parameters of deaf handball players.

## Methods

### Participants

The working group consists of players from the Turkish Men’s Deaf Handball National Team. A total of 30 (age: 23.7± ± 5.6 years; height: 176.5± ± 8.9 cm; weight: 77.3 ± ± 4.2 kg) deaf handball national team players voluntarily participated in this study. The inclusion criteria include having at least 5 years of sports history. The exclusion criteria for the study are that the participant has taken a break from the national team training camp due to any disability. All participants were required to sign a written informed consent form before the commencement of the current research. Volunteer participants were informed about the benefits and potential risks associated with the study. The study was conducted in accordance with the Declaration of Helsinki and with the participants’ signed consent forms. The lowest hearing loss rate among participants was 65 dB (decibels), the highest was 90 dB and above, and the average was 85 dB. Approval was obtained from the Afyon Kocatepe University Health Sciences Scientific Research and Publication Ethics Committee (Date and Number: 08.06.2023–187337).

### Experimental procedure

Before starting the research, the participants were provided with comprehensive information about the research design, and a detailed awareness was raised to ensure their participation in the research process. At this stage, players from the national deaf handball team who agreed to participate in the study were asked to review and sign the informed consent form. This step aims to ensure that participants are fully involved in the research process and join with conscious consent in accordance with ethical principles.

The general plan of the research is to implement it in four camp periods. Each camp period lasts approximately 10 to 12 days. The study covers a total period of 45 days. During this period, the daily training programme consisted of one session one day and two sessions the next day. The seventh day was a rest day for the athletes. Training continued on the following days with single and double sessions. The athletes went to bed at 11:30 p.m. and got up at 7:00 a.m. On days with a single session, training took place between 5:00 p.m. and 6:30 p.m. On days with double training sessions, the morning session took place between 9:00 and 10:30, and the evening session between 17:00 and 18:30. On days with double training sessions, basic motor skills were practiced in the fitness center in the morning. At the same time, sport-specific technical and tactical work was conducted during evening training sessions on both single and double training days. This diversity symbolizes the exposure of athletes to various training intensities, while simultaneously enriching the research results. As expected, participants were asked to complete a Borg scale, ranging from 1 to 10, to assess the burden of training on athletes via a Google survey. This subjective scale has helped us evaluate athletes’ training experiences more effectively. In addition, participants rated the following questions about sleep quality on a scale of 1 to 5 each day. These questions emphasize that sleep quality is a crucial variable considered within the scope of research to understand the effects of athletes on overall health and performance. This comprehensive method is designed to enhance the scientific integrity of the study and enable the meaningful analysis of data obtained from the participants.

### Training load monitoring

The internal loads of the athletes were determined using the RPE method. The 10-point Foster scale, developed by Foster et al. (2021), was used to monitor the RPE of the athletes after each training session [25]. After training, the question ‘How difficult was the training for you?’ was asked about 30 min later, and the athletes’ perceptions were evaluated. The athletes’ training times were recorded in minutes along with their RPE responses. Before the study, all athletes underwent a special training session to familiarize them with the RPE scale [10].

### Sleep quality monitoring

Participants were asked to complete an online Google Docs survey before 4:00 p.m. on their daily training day. The researcher instructed the participants to complete the questionnaire via Google Forms at 10:00 a.m. every day. Participants were asked to answer verbally if they did not complete the questionnaire by 3:30 p.m. on training days. Survey: How long did it take you to fall asleep (minutes)? How much did you sleep in total (how many hours)? How would you rate your sleep quality? How rested or refreshed did you feel when you woke up for the day? It contains four items. The survey questions used in our study were adapted from questionnaires previously used to monitor sleep quality in other studies [20, 26]. Participants answered the evaluation of each item on a 1–5 Likert scale. This questionnaire is designed similarly to the scales used in previous studies [20, 26].

*Question related to the sleep quality questionnaire.*

- How long did it take you to fall asleep (minutes)?
- How much did you sleep in total (how many hours)?
- How would you rate your sleep quality?
- How rested or refreshed did you feel when you woke up for the day?

### Statistical procedure

In the study, normality was tested using the Shapiro-Wilk test. After it was determined that the data did not show normal distribution, non-parametric tests were performed. Spearman correlation analysis was conducted to determine the relationship between Training Load and Sleep Quality. Differences in sleep quality and training load parameters across the blocks were analyzed using the Kruskal–Wallis test, as the data were nonparametric. When significant differences were found, Dunn’s posthoc analysis was conducted to identify which blocks differed significantly from one another. Eta square values were reported for effect sizes. η² values in the range of 0–0.009.009 were considered as insignificant effect sizes, 0.01–0.0588.01.0588 were regarded as minor effect sizes, 0.0589–0.1379 were considered as medium effect sizes, and values greater than 0.1379 were regarded as major effect sizes. The magnitude of the Spearman correlation is defined as: *r* < 0.1, insignificant; 0.1 < *r* ≤ 0.3, small; 0.3 < *r* ≤ 0.5, medium; 0.5 < *r* ≤ 0.7, large; 0.7 < *r* ≤ 0.9, huge, and *r* ≤ 0.9, almost perfect. The significance level was determined as *p* ≤ 0.05 in all statistical tests. All data processing steps and analyses were performed with the R programming language.

## Results

**Fig. 1:**
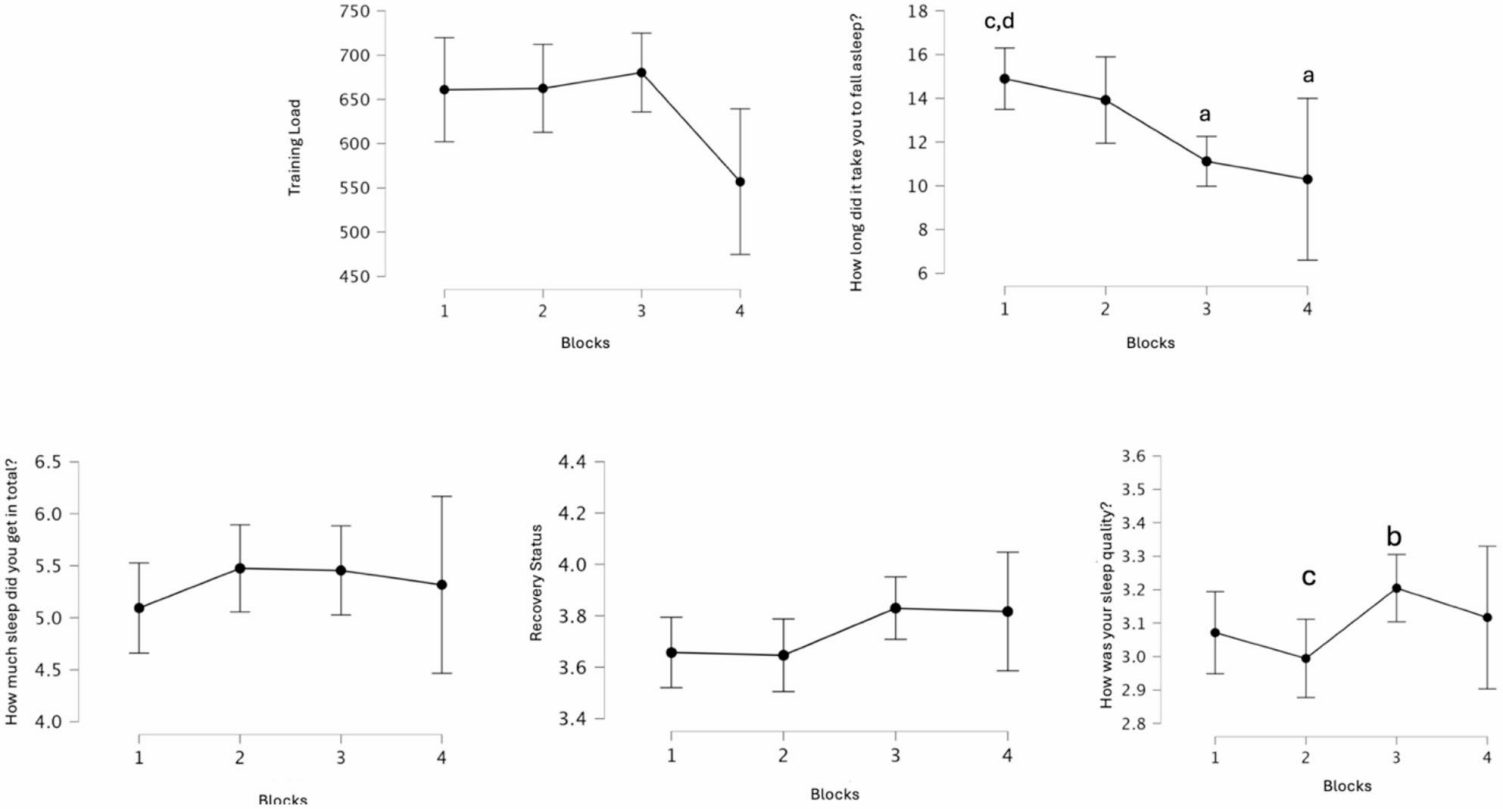
Post hoc comparative analysis showing differences between weeks; **A**: Different from the week in block 1; **B**: Different from the week in block 2; **C**: Different from the week in block 3; **D**: Different from the week in block 4

**Fig. 2:**
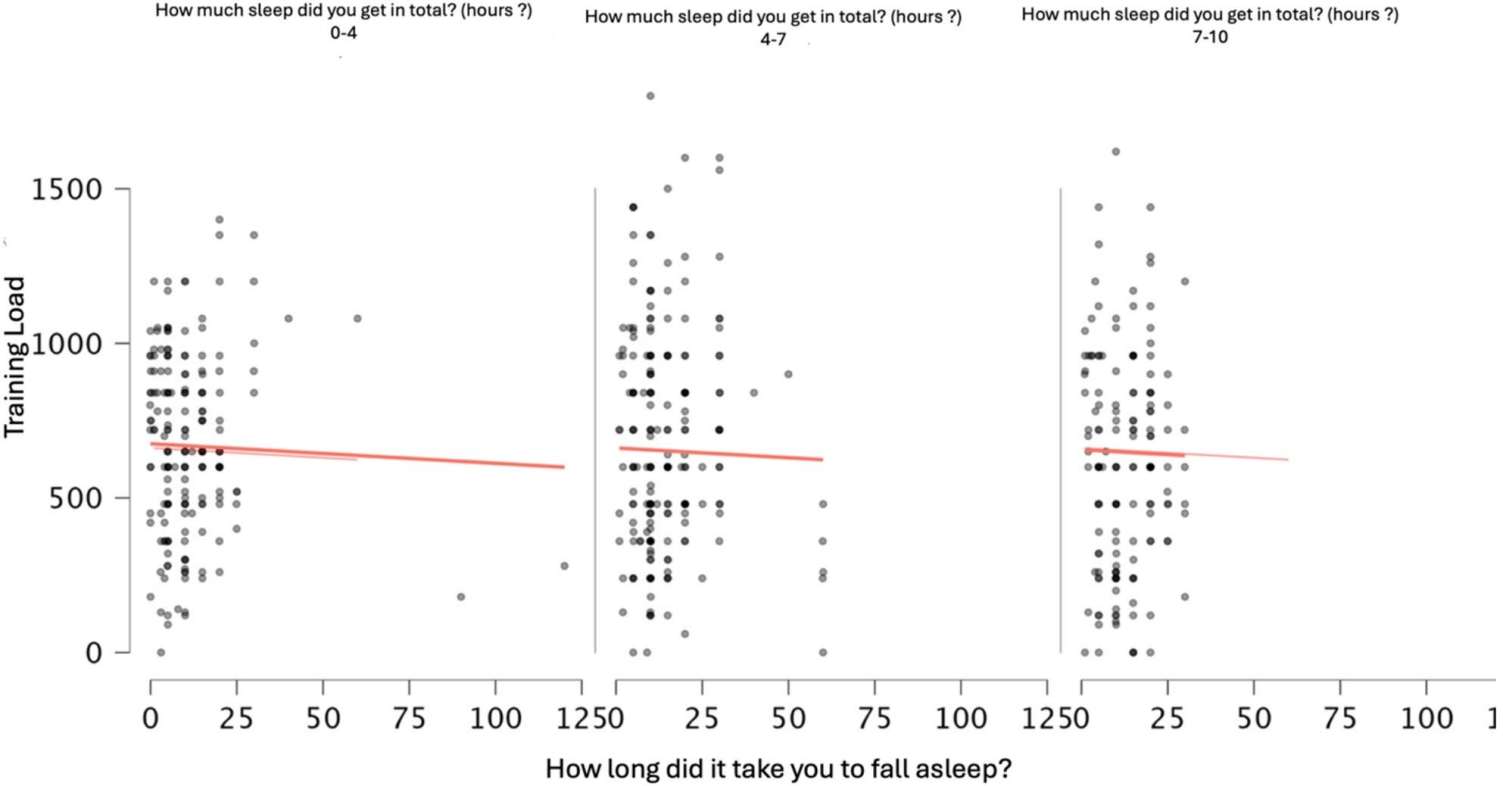
Descriptive values of Training Load, Total time asleep, and time to fall asleep

**Fig. 3:**
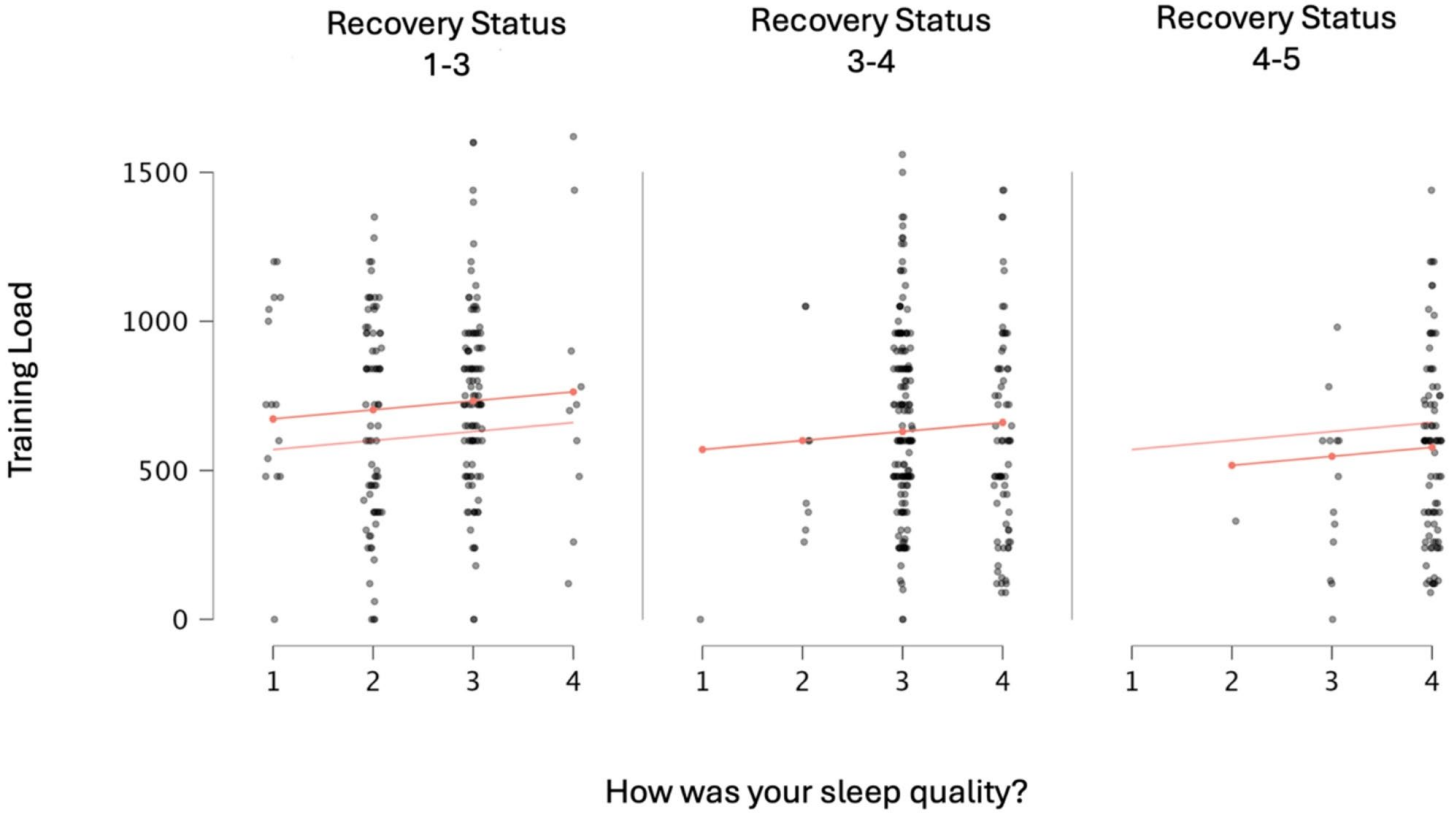
Training Load, Rest status, and sleep quality descriptive values

**Fig. 4:**
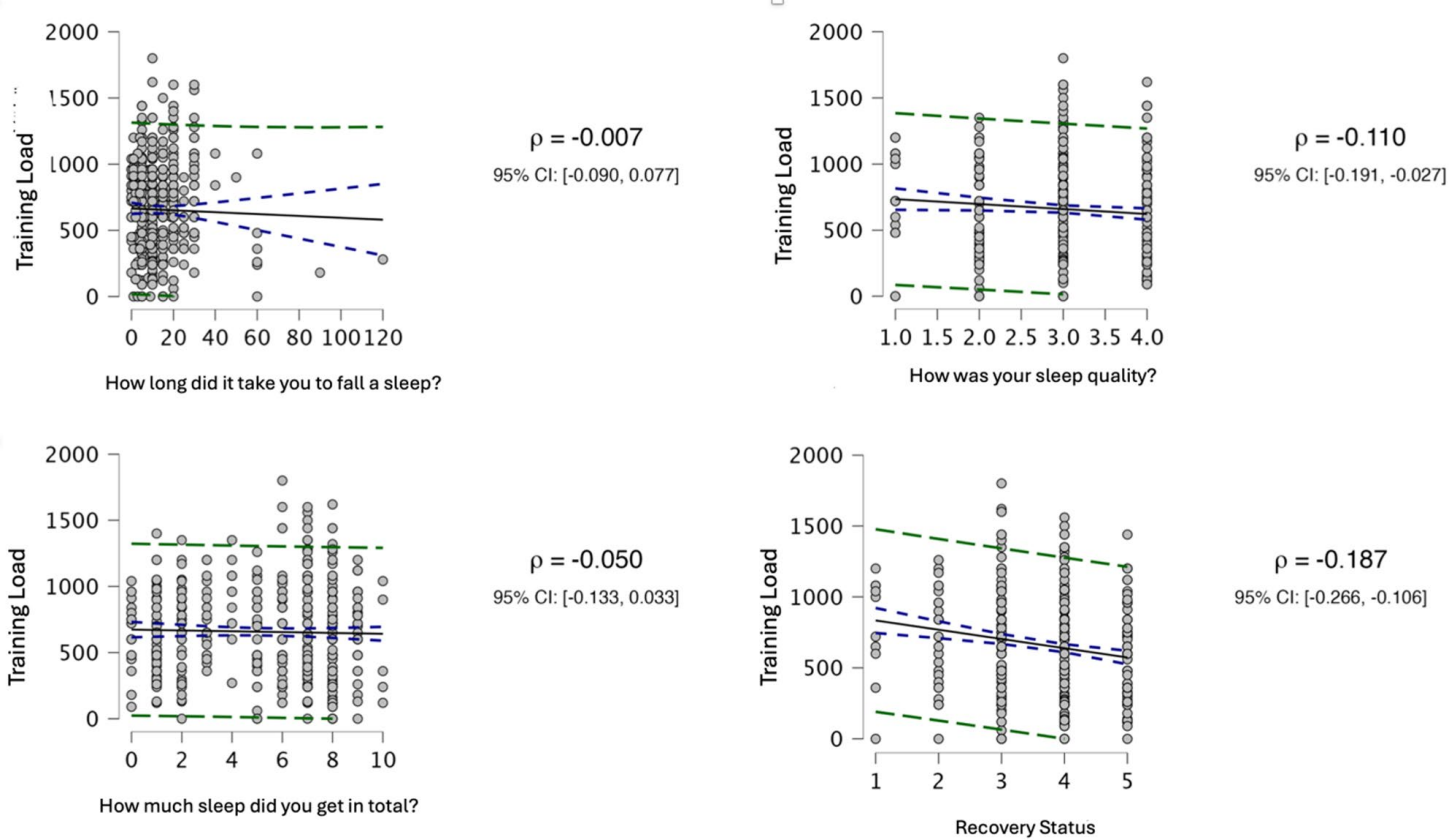
Relationship values between Training Load, time to fall asleep, sleep quality, total sleep time, and resting status

As a result of the analysis, statistically significant differences were found between training load and sleep quality across weeks, time to fall asleep across weeks, and sleep quality. Details of the post-hoc comparative analysis, showing the differences between weeks, are presented in Fig. 1. The descriptive values of all findings related to training load and sleep quality are shown in Figs. 2 and 3. As a result of the Spearman correlation analysis, no statistically significant relationship was found between the data in the study. A detailed analysis of the data is presented in Fig. 4. According to the findings of a one-way ANOVA conducted to determine the differences between the blocks, there was a statistically significant difference in the question “How long did it take you to fall asleep (in minutes). Details regarding the analysis results are provided in Table 1.

**Table 1:** Differences between blocks determined by the Kruskal– Wallis test

Situation	*P*	η²_p_
Training Load	0,061	0,008
How long did it take you to fall asleep (minutes)	0,001	0,057
How much did you sleep in total (how many hours)?	0,331	7.588 × 10^− 4^
How would you rate your sleep quality?	0,103	0,006
How rested or refreshed did you feel when youwoke up for the day?	0,217	0,003

## Discussion

This study aimed to examine the (i) relationships and (ii) weekly differences of training load and sleep quality parameters of deaf handball players. In this study, the training load and sleep quality of deaf handball players were recorded during the camp period. The analyses revealed statistically significant differences across weeks in both training load and sleep variables (sleep latency and sleep quality). Moreover, the Spearman correlation analysis showed no statistically significant correlation between sleep quality and training load values.

The volume and intensity of workouts can affect the recovery dynamics of athletes after training [2, 3, 27]. The intensity and volume of the workouts not only affect the recovery dynamics during the workouts, but can also activate many components during the workout [28–30]. The recovery dynamics after training are directly proportional to the intensity and volume put forth during training [28, 30]. While engaging in these activities, physiological responses to the stress created in the body emerge [7, 31]. It exhibits distinct physiological responses to changes in intensity and volume [3, 8, 9]. As the RPE values of the athletes’ efforts in training increase, many fatigue parameters increase at a corresponding rate [10, 25, 32]. This situation can also directly affect recovery strategies after activities [3, 9]. If the load of training on the organism is high, physiological responses can cause increased fatigue in athletes [3, 6, 10]. As a result of the increased training load, the recovery and rest needs of athletes may be higher [6, 8–10].

The dynamics of recovery after athletic activities may vary according to several variables [3]. Training loads can affect all physiological processes of athletes [3]. It can affect both performance outcomes in the following workouts and sleep quality [18–20]. In the literature, several studies have investigated the relationship between sleep and performance [18, 19, 22, 24, 33]. Research indicates that changes in training loads reduce sleep quality [18, 19]. When athletes are subjected to high training loads, they tend to fall asleep, and the duration of their sleep decreases [19, 20]. In our study, unlike other studies, no relationship was found between sleep quality and training load. It is thought that one of the biggest reasons for this may be the duration of our research.

In the present study, no significant correlation was observed between training load and sleep quality parameters. This finding may be attributed to the relatively stable training demands across blocks, as well as individual differences in sleep perception and adaptation to training stress. Previous research has suggested that while high training loads can negatively influence sleep, well-conditioned athletes may exhibit resilience to short-term fluctuations in load. Moreover, the specific characteristics of deaf soccer players, including enhanced visual communication demands and altered sensory processing, might influence sleep quality differently compared to hearing athletes. However, these interpretations should be considered with caution, given the study’s limitations, including the small sample size, subjective assessment of sleep, short observation period, and absence of a control group. Future studies incorporating objective sleep monitoring (e.g., actigraphy) and larger samples are warranted to validate these findings.

Although the 45-day observation period and the four training blocks provided valuable insights into short-term adaptations and fluctuations in players’ responses, they may not fully capture the long-term effects of accumulated training load and recovery dynamics. The relatively short duration was selected to ensure consistent environmental and competitive conditions during the in-season phase and to minimize potential confounding factors such as schedule disruptions, injuries, or tactical changes. Future studies with more extended monitoring periods are warranted to confirm whether these short-term responses reflect long-term adaptation patterns. Additionally, it is possible that our research group’s composition, which includes handball players, may have yielded different results compared to other studies. The findings of our study included data from different weeks during the camp period, with each week representing a distinct training block. The content of the workouts varied within the blocks. In our analyses to examine the differences between the blocks, we found statistically significant differences in the time it took to fall asleep. At the same time, it was observed that as the weeks of our study progressed, the time it took to fall asleep decreased. This is thought to be because, as the weeks progress, the cumulative fatigue of the training load on the athletes may increase, causing them to need more sleep and fall asleep more quickly. However, it is recommended that future studies be planned over a more extended period and that studies using biochemical parameters and different training load measurement methods be conducted.

Although the present study relied on subjective measures such as ratings of perceived exertion (RPE) and self-reported sleep quality, these tools are widely used and validated in field-based monitoring of athletes. Previous research has demonstrated that RPE provides a reliable and practical indicator of internal load and perceived fatigue in both training and competition settings. Similarly, subjective sleep assessments have demonstrated acceptable agreement with objective measures, such as actigraphy, particularly when daily monitoring in real-world environments is required. Nevertheless, the absence of objective indicators (e.g., actigraphy, biochemical markers, or neuromuscular tests) represents a limitation that should be addressed in future studies. Combining subjective and objective monitoring methods would provide a more comprehensive understanding of athletes’ fatigue and recovery states. These findings highlight the importance of tailored sleep management and recovery strategies for deaf athletes. Coaches and sports practitioners can utilize this information to optimize training schedules, implement individualized recovery protocols, and develop effective communication methods that take into account sensory differences. By understanding the unique factors influencing sleep and recovery in this population, teams can enhance performance, reduce injury risk, and support overall athlete well-being.

## Conclusion

There was no statistically significant relationship between the sleep quality and training load values of handball players. There are statistically significant differences between sleep quality and training load values between weeks. As a result of the analysis, statistically significant differences were found between training load and sleep quality across weeks, as well as between time to fall asleep and sleep quality across weeks. Sports scientists and practical practitioners can take action by considering the findings of our research when planning the training loads and sleep quality values for their athletes.

## Data Availability

Data is available to the corresponding author upon reasonable request
